# Identifying Molecular Chechkpoints for Adventitious Root Induction: Are We Ready to Fill the Gaps?

**DOI:** 10.3389/fpls.2021.621032

**Published:** 2021-03-05

**Authors:** Dolores Abarca

**Affiliations:** Department of Life Sciences, University of Alcalá, Alcalá de Henares, Spain

**Keywords:** adventitious rooting, auxin, epigenetic regulation, meristem regeneration, non-cell autonomous signaling, sugar signaling

## Abstract

The molecular mechanisms underlying *de novo* root organogenesis have been under intense study for the last decades. As new tools and resources became available, a comprehensive model connecting the processes and factors involved was developed. Separate phases that allow for specific analyses of individual checkpoints were well defined. Physiological approaches provided information on the importance of metabolic processes and long-distance signaling to balance leaf and stem status and activation of stem cell niches to form new root meristems. The study of plant hormones revealed a series of sequential roles for cytokinin and auxin, dynamically interconnected and modulated by jasmonic acid and ethylene. The identification of genes specifying cell identity uncovered a network of sequentially acting transcriptional regulators that link hormonal control to cell fate respecification. Combined results from herbaceous model plants and the study of recalcitrant woody species underscored the need to understand the limiting factors that determine adventitious rooting competence. The relevance of epigenetic control was emphasized by the identification of microRNAs and chromatin remodeling agents involved in the process. As the different players are set in place and missing pieces become apparent, findings in related processes can be used to identify new candidates to complete the picture. Molecular knobs connecting the balance cell proliferation/differentiation to hormone signaling pathways, transcriptional control of cell fate or metabolic modulation of developmental programs can offer clues to unveil new elements in the dynamics of adventitious rooting regulatory networks. Mechanisms for cell non-autonomous signaling that are well characterized in other developmental processes requiring establishment and maintenance of meristems, control of cell proliferation and cell fate specification can be further explored. Here, we discuss possible candidates and approaches to address or elude the limitations that hinder propagation programs requiring adventitious rooting.

## Introduction

The ability to form roots was essential for plants to colonize the land. Evolutionary studies suggest that they appeared more than once in the land plant lineage, recruiting components of signaling pathways involved in the establishment and maintenance of stem cell niches (Xu et al., 2016; Mhimdi and Pérez-Pérez, 2020). As land plants evolved to adapt to a variety of developmental and environmental challenges, root architecture diversified and adventitious roots (AR) with new functions were added to the root systems of different plant groups (Gonin et al., 2019).

The possibility to induce ectopic root formation from cuttings is widely used for vegetative propagation. Understanding the mechanisms underlying adventitious rooting is essential to expand our knowledge of plant regeneration processes and can provide tools to improve propagation for commercial or ecological purposes. Plant scientists have studied it for centuries, and the complexity of new discoveries has paralleled scientific advances. The use of experimental systems based on model plant species such as cut or intact *Arabidopsis* hypocotyls, *Arabidopsis* leaf explants, or petunia shoot tip cuttings, has proven invaluable to test hypotheses and establish connections between signaling pathways (Díaz-Sala et al., 2002; Sorin et al., 2005; Chen et al., 2014; Druege and Franken, 2019). Research on recalcitrant species, on the other hand, involves complementary approaches that use loss of competence as a tool to identify hubs for hormonal and developmental control networks (Abarca et al., 2014; Aumond et al., 2017; Sun et al., 2019; Guan et al., 2020, Velada et al., 2020). In the last decades, new techniques and resources provided many answers and helped to understand what is common and what is unique in different plant groups, allowing for a comprehensive model that can be used as a scaffold for further research (De Klerk et al., 1999; Da Costa et al., 2013; Druege et al., 2014; Druege et al., 2016; Xu, 2018; Jing et al., 2020).

Current knowledge of the basic biochemical, genetic, cellular, physiological, and environmental processes involved in *de novo* root organogenesis can provide clues to identify bottlenecks for vegetative propagation and introduce quantitative improvements (Druege, 2020). However, woody species that show a sharp age-related decline in adventitious rooting capacity (Abarca and Díaz-Sala, 2009; Pizarro and Díaz-Sala, 2019; Valladares et al., 2019) may need a qualitative approach to unlock signaling pathways that successfully elicit the formation of new root meristems in most herbaceous plants. Since the main regulatory elements seem to be widely conserved (Abarca et al., 2014; Álvarez et al., 2018; Gonin et al., 2019; Mhimdi and Pérez-Pérez, 2020), the possibility that specific checkpoints are under distinctive control mechanisms in recalcitrant species must be considered. Here, we integrate known players, identified using different experimental systems, into the scaffold of a general model, discuss how findings in related processes can offer new insights into the complex interactions between regulatory networks and propose a new experimental approach to test potential candidates to improve adventitious rooting from cuttings in propagation programs.

## Adventitious Root Induction: A Surge of Hormonal and Metabolic Changes…

**Figure 1:**
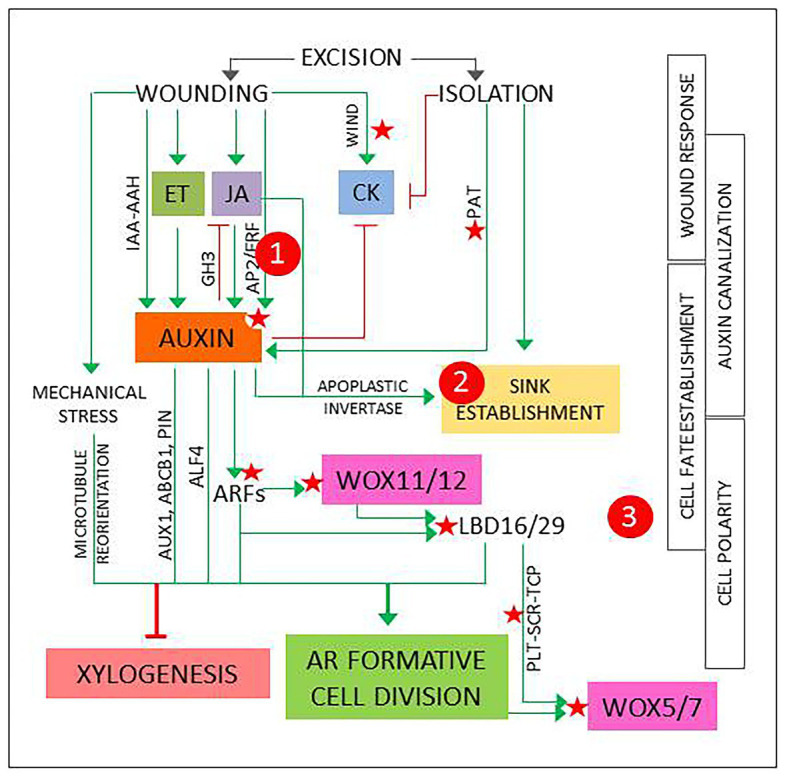
The first events and players in adventitious root induction, described in different experimental systems as referred in the main text and integrated in a general model. Green arrows and red lines represent positive and negative regulation, respectively. Red stars mark points of known epigenetic regulation. 1, 2, and 3 signal possible checkpoints to identify new candidates, as explained in Figure 2.

According to the accepted model, AR development starts with a 24–48 h induction phase. It involves a process of fate reassignment in competent cells that will be reprogrammed to define new root founder cells and ends with a formative cell division (De Klerk et al., 1999; Druege et al., 2016; Xu, 2018; Jing et al., 2020). This signals the start of the initiation phase, when the new meristem is defined, and is followed by an expression phase that leads to AR emergence. The process can last days to weeks depending on the type of explant, the physiological, and developmental status of the mother plant and the environmental conditions (Xu, 2018; Druege 2020). The signaling pathways set in motion during the induction phase start a chain of events that will determine the overall success and will be the main focus hereinafter. Figure 1 presents a model that integrates basic events and players that shape the induction phase and have been identified and analyzed using a variety of experimental systems.

In the first 2 h, transient hormone changes predominate. In shoot or hypocotyl cuttings, the excision elicits wound response signaling pathways that result in a quick, transient increase in jasmonic acid (JA) and ethylene (ET; Druege et al., 2016). Another fast, local wound response described in *Arabidopsis* is the induction of *WOUND INDUCED DEDIFFERENTIATION* (*WIND*) genes, encoding APETALA2/ETHYLENE RESPONSE FACTORs (AP2/ERFs) that promote B-type *ARABIDOPSIS* RESPONSE REGULATOR (ARR)-mediated cytokinin (CK) responses and have been shown to induce callus formation in *Brassica napus*, tomato, and *Nicotiana tabacum* (Iwase et al., 2011). *WIND* genes have been proposed to have a role in reactivation of cell proliferation (Bustillo-Avendaño et al., 2018).

The initial reaction is followed by a local increase in auxin concentration and sensitivity (Figure 1). Genes involved in auxin biosynthesis and signaling are up- or downregulated (Druege et al., 2014), possibly due to a combination of direct and indirect wound responses mediated by JA and ET (Lakehal and Bellini, 2019; Zhang et al., 2019). A recent report describes two JA- and wound-responsive *AP2*/*ERF* genes that mediate auxin biosynthesis at the early phases of AR development (Ye et al., 2020). In addition, local release of conjugated auxin by IAA-amino acid hydrolases (IAA-AAH), as described for petunia cuttings, can contribute to the increase of bioactive IAA (Druege et al., 2019). Finally, as root-directed polar auxin transport (PAT) is interrupted, auxin from the shoot will accumulate at base of the cutting (Druege, 2020; Jing et al., 2020).

The local auxin accumulation will induce genes encoding GRETCHEN HAGEN3 (GH3) acyl-acid-amide synthetases that generate inactive JA conjugates, contributing to return JA to basal levels (Gutiérrez et al., 2012; Druege et al., 2014; Vielba et al., 2016). As a consequence of root excision, CK levels are gradually reduced. Complex interactions between auxin, CK, and JA signaling pathways, dynamically interconnected, promote high auxin/CK balances and low concentration of bioactive JA (Druege et al., 2016; Lakehal and Bellini, 2019).

In parallel with hormonal changes, metabolic adjustments result from the loss of water and mineral nutrients supplied by the roots, combined with auxin and JA-mediated induction of apoplastic invertase that promotes the establishment of a sink competing with shoot meristems and young leaves (Druege et al., 2019). This reallocation of metabolic resources can be interpreted as component of the wound response, temporarily needed to support new growth and ensure wound closure, combined to the effect of auxin accumulation at the base of the cutting. However, the resulting changes in local sucrose concentration can elicit signaling pathways that link metabolic and hormonal control of plant development (Smeekens et al., 2010; Lastdrager et al., 2014). The possible relevance of these events is discussed below.

## …Is Followed by a Chain of Auxin Responses That Lead to Root Founder Cell Specification

The rest of the induction phase is dominated by auxin responses in cells located at, or next to, the vascular cylinder. Depending on the type of explant, AR source cells can be pericycle-like, (pro)cambium or vascular parenchyma cells, usually located next to the xylem poles (Jing et al., 2020). What these cells have in common is that they retain, or can recover, proliferative capacity in response to specific stimuli. In fact, cells at the vascular cylinder next to xylem poles seem to have a high versatility, since they are involved not only in regeneration and vascular development, but also in a variety of developmental processes related to biotic interactions, such as nodulation or cyst formation in nematode infections (Crespi and Frugier, 2008; Vieira and Gleason, 2019).

In vascular development, (pro)cambial cells near xylem poles form xylem cells. This process requires a previously established auxin gradient that will induce a specific program for xylem cell specification, followed by an asymmetric, periclinal cell division to form a xylem cell precursor (Milhinhos and Miguel, 2013; Kajala et al., 2014). The formation of a new root meristem requires to induce a similar process in similar cells with a different result, suggesting that AR source cells need to acquire a root founder precursor identity prior to the first cell division. AR source cells undergo a reprogramming process controlled by auxin-responsive genes encoding AUXIN RESPONSE FACTORs (ARFs). Induction of *WUSCHEL-RELATED HOMEOBOX11* (*WOX11*) and its functional homolog *WOX12* is followed by upregulation of *LATERAL ORGAN BOUNDARIES DOMAIN16* (*LBD16*) and *LBD29*. These changes can be used as markers for cell fate transition: at this point, AR source cells are ready for a formative, typically anticlinal cell division that will form the root meristem founder cells. Non-cell autonomous signaling from one of the daughter cells will induce the expression of *WOX5* and *WOX7*, specific quiescent center (QC) markers, in the other one, while repressing the expression of *WOX11*/*12* and *LBD16*/*29*. This signals the transition to root primordium cells (Liu et al., 2014; Jing et al., 2020).

The choice between AR induction and xylogenesis requires a specific combination of biochemical, positional, and mechanical signals (Figure 1). Positive and negative epigenetic markers have been identified in *Arabidopsis* for most of the components of the AR auxin signaling pathway (Jing et al., 2020). In addition, *miR160* and *miR167* modulate the levels of specific ARFs that regulate AR induction in *Arabidopsis* intact hypocotyls (Gutiérrez et al., 2009) and have been related to adventitious rooting in a variety of plant species (reviewed in Gleeson et al., 2014). Genes encoding transcriptional regulators PLETHORA1/2 (PLT1/2), SCARECROW (SCR), and TEOSINTE-BRANCHED CYCLOIDEA PCNA (TCP), that establish and maintain QC in embryogenesis and lateral root formation, are induced in the root founder precursor cells (Jing et al., 2020).

Positional information can derive from auxin canalization. In the first 24 h of the induction phase, a local auxin gradient is established that provides the positional signals required to direct the ensuing processes. Up- and downregulation of genes encoding specific auxin influx and efflux carriers, such as *AUXIN1* (*AUX1*), *ATP-binding cassette transporter1* (*ABCB1*), or members of the *PIN-formed* (*PIN*) family, suggest that auxin is canalized to AR source cells *via* PAT (Druege et al., 2014; Jing et al., 2020). In addition, chemical disruption of PAT during the first 24 h reduces drastically AR induction (Díaz-Sala et al., 2002; Pacurar et al., 2014). Furthermore, the capability to canalize auxin transport to cambial cells next to the xylem poles has been associated to adventitious rooting competence (Abarca et al., 2014).

The orientation of the cell division plane relies on a previous cell polarization guided by the cytoskeleton, which links intracellular signals, the plasma membrane, and mechanical stimuli on the cell wall (Díaz-Sala, 2019; Druege et al., 2019). Local pressure changes caused by wounding could be read as specific signals for cell wall remodeling and microtubule reorientation. As a consequence, the integration of biochemical, positional, and mechanical signals would promote an anticlinal cell division in the AR source cells.

## Identifying Molecular Checkpoints for Adventitious Rooting Competence

Once a general model is established and well-conserved regulatory pathways are identified, the problem of limiting factors in recalcitrant plants can be addressed. Two obvious questions arise: first, low-rooting cuttings are often able to produce a low number of AR after long incubations, frequently from callus-like cell masses. This could be an indirect effect of the common signaling pathways found in calli and root meristems (Sugimoto et al., 2010) and suggests that there is an stochastic component in the process, but it usually results in poor vascular connections (Da Costa et al., 2013). The obvious implication is that prolonged cell proliferation previous to AR induction is not a reliable strategy to optimize propagation programs. The focus should, therefore, be on the induction phase.

The second question is whether we can expect a common limiting factor for all cases. Recalcitrance is typical of woody plants, which frequently suffer an age-related loss of regeneration capacity. This can often be observed in relatively young plants, even before maturation produces detectable changes. Secondary growth is a tempting candidate, implying that the adventitious rooting capacity can decrease after the procambium-cambium transition, when rings of secondary xylem may hinder the formation of wound-induced mechanical gradients leading to microtubule reorganization before the first formative division (Pizarro and Díaz-Sala, 2019). This can result in a decrease or a delay in AR formation, but it does not explain cases such as genotype differences in cuttings of the same age, the formation of AR in excised branches of riparian woody species or age-related decline in explants from herbaceous model species. The conclusion is that it is safer to assume that more than one factor could be relevant to understand adventitious rooting competence.

### Age-Related Chromatin Remodeling

An obvious candidate is epigenetic control of gene expression. Development- and stress-related changes in the epigenetic landscape are well-known, so it is no surprise that key components of the adventitious rooting auxin signaling pathway are under epigenetic control (Figure 1). Epigenetic regulation is also known to control vegetative and reproductive phase changes. In *Arabidopsis*, chromatin remodeling promotes an age-related decrease in the levels of *miR156*, which targets *SQUAMOSA PROMOTER BINDING PROTEIN-LIKE* (*SPL*) genes. The targeted *SPL*s control the expression of *AP2*-like genes that contribute to maintain the vegetative meristem identity. Reduced *miR156* levels upon phase transition allow for an SPL-induced decrease in *AP2*-like gene expression (Xu et al., 2016). Similar processes have been described in herbaceous and woody species (Wang et al., 2011; Curaba et al., 2014), so it seems to be a well-conserved mechanism for phase-change related reprogramming of gene expression.

**Figure 2:**
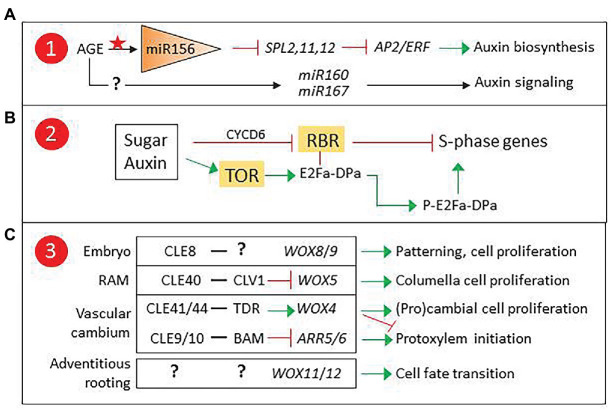
Proposed checkpoints for age-related decline in adventitious root induction. **(A)** Epigenetic control of *miR160*/*167* could modulate auxin signaling. **(B)** TARGET OF RAPAMICINE (TOR) developmental regulation could affect sugar and auxin control of the G1-S transition. **(C)** CLAVATA3/EMBRYO SURROUNDING REGION-RELATED (CLE) peptides and their receptor-like kinases (RLKs) are involved in the control of apical and vascular meristem stem cell proliferation *via* non-cell autonomous signaling, and could regulate *WOX11*/*12* expression in adventitious roots (AR) founder cells. 1, 2, and 3 refer to Figure 1. ARR5/6, Type A *Arabidopsis* Response Regulators 5/6, modulators of cytokinin signaling; BARELY ANY MERISTEM (BAM), CLAVATA1 (CLV1), and TDIF receptor (TDR) are RLKs.

Interestingly, *miR390*, which targets *ARF* genes involved in lateral root induction (Curaba et al., 2014), is also involved in phase transition-related *SPL* regulation. In a recent report, three *miR156*-targeted SPLs were found to repress *AP2/ERF* genes involved in wound-induced auxin biosynthesis, establishing a clear connection between age-related epigenetic control and competence for adventitious rooting (Ye et al., 2020). Since the expression of adventitious rooting-related *ARF* genes is epigenetically regulated, it is tempting to propose that a similar mechanism could modulate *miR160*/*miR167* levels (Figure 2A).

Another possible candidate for age-related epigenetic control is *ABERRANT LATERAL ROOT FORMATION4* (*ALF4*), a gene encoding a regulator of SCF^TIR1^, an E3 ligase complex involved in auxin signaling (Bagchi et al., 2018). In lateral root formation, *ALF4* expression is involved in auxin-induced specification of founder cells and has been proposed as a marker for cell proliferation capacity (Dubrovsky et al., 2008; Kajala et al., 2014). Mutant analysis suggests that it could play a similar role in AR induction (Jing et al., 2020), signaling it as a candidate to integrate hormonal and developmental control of adventitious rooting competence, where epigenetically controlled *ALF4* expression could result in a developmentally controlled modulation of auxin responses in AR source cells.

### Sugar Signaling and Cell Cycle Control

A second factor to consider is wound-induced metabolic changes. Cells next to the excision receive a combination of wound and auxin signals, together with a transient decrease, followed by an increase, in sucrose concentration (Ahkami et al., 2009). These signals are integrated in the AR source cells, which will start a reprogramming process previous to the formative division. Both auxin and sugars are known to regulate the cell cycle (Figure 2B). The G1-S transition is controlled by RETINOBLASTOMA-RELATED (RBR), a protein that regulates the balance between cell proliferation and differentiation in meristems (Borghi et al., 2010; Gutzat et al., 2012; Harashima and Sugimoto, 2016). RBR binds and inactivates E2Fa-DPa, a transcription factor complex that induces the expression of S-phase genes. Auxin and sugars can counteract RBR by inducing *CYCLIND6* (*CYCD6*), which promotes RBR phosphorylation, releasing the E2Fa-DPa complex. Alternatively, they can induce the expression of *TARGET OF RAPAMICINE* (*TOR*), encoding a kinase that phosphorylates E2Fa, releasing it from RBR and thus enabling the induction of S-phase genes (Xiong and Sheen, 2014).

RBR and TOR connect metabolic and auxin signaling in cell cycle regulation. TOR has been reported to regulate AR induction in *Arabidopsis* and potato (Deng et al., 2017) and participate in meristem size regulation by controlling cell proliferation (McCready et al., 2020). In addition, TOR inhibition has been reported to produce a decrease of DNA methylation affecting genes involved in hormone signaling, suggesting a role in chromatin remodeling (Zhu et al., 2020). Overall, experimental evidence signals TOR as a candidate for adventitious rooting competence.

### Non-cell Autonomous Peptide Signaling in the Control of Stem Cell Proliferation

A third point of control to consider is the regulation of *WOX11/12* expression. *WOX* genes are involved in cell-to-cell communication to modulate stem cell proliferation and differentiation. They are essential players in plant developmental processes involving root meristem specification and maintenance (Figure 2C). They are also important in the control and maintenance of vascular stem cells and have central roles in the regulation of xylem and phloem cell specification (Fletcher, 2020). In addition, they participate in biotic interactions such as nodulation and have been recruited by pathogens such as cyst nematodes to induce the formation specific structures (Whitewoods, 2021). Non-cell autonomous signaling to control *WOX* gene expression is carried out by CLAVATA3/EMBRYO SURROUNDING REGION-RELATED (CLE) peptides that are secreted by neighboring cells and recognized by specific receptor-like kinases (RLKs). CLE-RLK pairs have been described for most *WOX* genes and other regulators of stem cell proliferation (Whitewoods, 2021).

*WOX11/12* are targeted by the Polycomb repressive complex 2 (PRC2). In the first hours after excision, repressive marks are removed (Jing et al., 2020). *WOX11/12* expression is then induced in the AR source cells and is subsequently restricted to cells surrounding the AR founder cells after the first formative division. Whether the mechanism underlying the selective *WOX11*/*12* repression in AR founder cells is based in cell-to-cell communication is unknown. The identification of a CLE-RLK pair targeting *WOX11/12* would offer new clues on the role of non-cell autonomous signaling in adventitious rooting competence.

## New Experimental Approaches for Functional Analysis of Multiple Candidates

The potential candidates for adventitious rooting competence proposed here are likely to represent just the tip of the iceberg. A process that requires the coordination of dynamically interconnected regulatory networks, depends on epigenetically controlled intermediates and relies on mechanical and biochemical signals for cell-to-cell and long distance communication, presents too many weak spots to allow for a simple answer. In many cases, unavoidable developmental factors, such as secondary growth or a lower PAT induction that reduces channeling and leads to an ambiguous response to auxin, can hardly be overcome using simple methods. Moreover, the potential complexity of the problem can hinder the design of experimental approaches to test new candidates.

Regeneration recalcitrance is a general problem when plant biotechnology protocols are transferred to non-model plants. In addition to clonal propagation *via* shoot/root regeneration or somatic embryogenesis, it seriously limits the success of transformation and gene editing programs, especially in species that lose the ability to regenerate early in development. In many important herbaceous and woody species, only immature embryos can be used to obtain proliferative masses that can regenerate new plants (Ishida et al., 2007; Lelu-Walter et al., 2013). A recent report proposes an innovative approach to overcome recalcitrance *via de novo* induction of meristems (Maher et al., 2020). The method is based on the transient expression of genes using co-cultivation (*in vitro*) or perfusion (soil-grown plants) of *Agrobacterium tumefaciens* cultures. It has been successfully used to test combinations of candidate genes for shoot regeneration in *Arabidopsis*, *Nicotiana benthamiana*, tomato, potato, and grape plants, using seedlings or mature plants. In general, the best results were obtained by combining *WUSCHEL2* (*WUS2*) with either *SHOOT MERISTEMLESS* (*STM*) or *ISOPENTENYL TRANSFERASE* (*IPT*), candidates selected for their roles in the maintenance of undifferentiated cells in the meristem (Chang et al., 2020).

A similar approach could be used to induce *de novo* root organogenesis. Combinations of well-known regulators involved in the establishment of root meristems, applied to high- and low-rooting cuttings, could provide a shortcut for qualitative and quantitative analyses of new candidates and offer new insights into the developmental control of adventitious rooting competence.

## Conclusion

A basic model of the events in *de novo* root organogenesis, resulting from the combined efforts of researchers using different experimental approaches, can be used to establish possible connections with related processes. Genes involved in epigenetic control of gene expression, metabolic control of the cell cycle, or non-cell autonomous signaling emerge as potential players in essential checkpoints. These and other candidates need to be studied to prove their relevance in the age-related regulation of adventitious rooting competence. Newly developed tools for *de novo* induction of shoot meristems can be adapted for this purpose, opening the possibility to perform quick tests not requiring the use of mutants or transgenic plants for functional analyses.

A protocol to induce root meristems using transient expression of suitable developmental regulators would prove invaluable as a tool to understand the mechanisms underlying adventitious rooting. In addition, it could be used as a shortcut to improve the efficiency of propagation programs for commercial or ecological purposes.

## Data Availability Statement

The original contributions presented in the study are included in the article/supplementary material, further inquiries can be directed to the corresponding author.

## Author Contributions

The author confirms being the sole contributor of this work and has approved it for publication.

### Conflict of Interest

The author declares that the research was conducted in the absence of any commercial or financial relationships that could be construed as a potential conflict of interest.
